# The Peace

**Published:** 1856-07-01

**Authors:** 


THE JOURNAL
OF
PSYCHOLOGICAL MEDICINE
AND
MENTAL PATHOLOGY.

JULY 1, 1856.

### Part First.

### Original Communications.

### Art. I.—THE PEACE.

From the eminent point of view which the happy consummation of peace has led us to, we may survey at our leisure the condition of the European powers. Warned by the past, we look upon the present state of the world with a cautious, as well as a critical eye. According to our conception, the study of *Mind*, in its broad physiognomy of nations and dynasties, of religion and civilization, is the highest point in the study of psychology. In the mental diseases of individuals, we are apt to lose sight of those great intellectual revolutions that break up the harmony of mankind, and involve the individual in the general ruin or disturbance of the whole. Thus, epidemic diseases sweep over wide portions of the earth, like the unchartered winds, and mock at the precautions of legislative quarantine, the rigid performance of *pratique*, and the *cordon sanitaire* of military boundaries. So, likewise, the mental phenomena seldom, if ever, appear in solitary cases, or, if they seem to do so, it is because we are not sufficiently aware of what is going on beyond our immediate sphere of vision, so as to perceive the extensive class of maladies to which they belong, and of which they are only isolated instances. Hence it is that we are so frequently staggered by crimes of the same nature developing themselves simultaneously, or coming, one after another, in different persons and widely separated localities. It is because they are the effect of vast moral changes, in operation over vast portions of the world, originating in occult, but by no means inexplicable causes. The true spirit of mental philosophy pre-eminently consists in understanding these great epochal psychological variations of the moral atmosphere.

It is no longer possible for this country, nor for any other, to stand isolated and apart from the rest of the civilized world. It is not possible, either, for statesmen or philosophers to act or think as if their own country were the only one that deserved their attention and interest. No nation can any longer pretend to the narrow and exclusive policy of exalting itself at the expense of all others, and of putting itself forward as the model republic, kingdom, or empire, for the rest to copy and work by. That day has passed away, we hope, for ever. The family of mankind are becoming one in thought and feeling; the period of slavery has virtually, if not actually, expired. Our interests are one. Mountains and seas, climates and hemispheres, may mark us off from each other in the many-coloured map of the universe; but our minds no longer recognise the real or artificial barriers between countries, nor the distance of space, nor the varieties of language, nor the peculiarities of manners, nor the difference of creed. It is useless to quarrel about a few hundreds of leagues of territory more or less, and worse than useless to quarrel about faith, which, if it do not amend the morals and correct the heart, is nothing better than an empty sound.

The trials and contests of the last fifty years have brought along with them their own dearly-bought experience, and, it must be added, with a velocity more than equal to a hundred and fifty years in any previous epoch of history. The nations have tried their strength and have failed. They have tottered on their slippery foundations: some of them have crumbled into nothing, while others have literally fallen to pieces. Europe has grown grey in feudalism, warfare, and theological disputes. America, liberal, independent, and young, has arisen out of the dissensions of her tenacious ancestors. *Our* prejudices are *her* freedom. On the other side, the Eastern populations have dwindled away into nothingness, abject slavery, and insignificance. China has potentially fallen; India is the *spolia opima* of Great Britain; and France, after oscillating violently between the two extremes of anarchy and despotism, has, for the present, settled down into a modified imperialism; Germany is still a huge mass of undigested fragments; Austria vacillates between Italy, Hungary, and St. Petersburg; while Russia, who only two years ago was soaring aloft, and motionless like an eagle on the wing, ready to swoop down on the first tempting prey that came within sight of her piercing eye, has fallen, and now lies fluttering at the feet of the two successful marksmen whose well-aimed rifles have brought her to the ground.

Russia has reached a crisis the most momentous to her existence. Situated both in Europe and Asia, she is intimately concerned in the well-being of either continent. Her Asiatic or Mongolian element need not distress her; for, though much too visible to escape detection, it just serves to impart to her that imposing air of superiority and dread so essential to what she has always aimed at becoming—the empress of the world. The testament of Peter the Great, apocryphal though it be, is nevertheless the index of the Muscovite temper; and the aggressive acts of the Czars have at least borne tangible evidence to the probability of its truth. Profiting by a lucky moment, says its fourteenth article, with a large army on land, and fleets at Archangel, in the Baltic and the Black Seas, the Mediterranean may be seized on, France invaded, and Germany subdued: these points gained, and the rest of Europe is ours. Late events are a practical comment on the reality of this *supposed* will of the Czar Peter. A serious inconvenience within the heart of Russia herself has, however, checked the earnestness with which she proposed to secure her conquests,—it is her religion, which retains too many traces of superstition and formalism ever to allow of her adopting any freedom of action in her efforts at political advancement. Peter the Great saw this important obstacle before him at the commencement of his reign. One of his first blows was aimed at the clergy, whose popular influence was incompatible with his own supremacy; and he fancied that with a stroke of this bold kind everything else would bend before him. He changed the Oriental style of dress for the Western—compelled his subjects to wear the frockcoat, and to shave their beards. But acts of tyranny of this childish sort cannot change a whole people at once; and Russia has not yet been able to coalesce with the Western Powers, nor to enter into the *universal* spirit of the age with which the rest of Europe has been so long and deeply imbued. She still remains intact and alone, swallowed up in the vastness of her boundless wastes; nor have her people manifested the influence of Christianity, in the plainest meaning of the word: for we must distinguish between the power of religion over the man, and the predilection of the man for his own religion. The one is a formality, the other a principle. They are two distinct things. It is one thing to observe a fast, or to die for a sacred image, and to carry a picture round the ramparts of a besieged fortress, for the purpose of inspiring or preserving devotion; but it is another thing to experience *that* Christianity which renders both the individual and his nation susceptible of the highest degree of virtue, science, and civilization.

Nevertheless, the Russians are an eminently brave nation—

kind-hearted, intelligent, hospitable, ingenious, and eloquent. Their language is said to be almost devoid of *patois*, or provincialism, from which so few languages are exempt. They are enterprising, fruitful in resources, and patient—crafty and diplomatic. After the defeat of Narva, Peter the Great was not in the least discouraged: "*Je sais bien*," was his cool remark on first learning the news—"*Je sais bien que les Suédois nous battront long temps, mais ils nous apprendront enfin à les battre!*"—a spirit of diplomacy from which we may do well to take a warning on the conclusion of the *present* peace—*quo tandem?*

The faults of Russia belong to her antique, if not antiquated, form of government, which was Tartar, as much as to her aggressive mode of civilization, which is intensely Russian; and her prejudices and government, both of them dating from the darkest epoch of the world, have not yet been reformed by the just demands of her people, nor remodelled by amalgamation with elements external to herself. The Russian sees his own fate in that of his Czars. With the exception of the late Emperor, Nicholas, their reigns have seldom exceeded thirteen years, while the average reigns of other European monarchs is about twenty-five; and as his Emperors have disappeared, no one scarcely knows how, so he himself disappears from his home, as a conscript or an exile, never more to return to his family hearth! In order to reach the level of general civilization, knowledge, popular freedom, and enlightened administration, a crisis, such as the present, was indispensably necessary to the very existence of "all the Russias." She could not advance by means of her own inherent vitality; she could not stand still while the rest of the world was advancing; and to recede was a national decease. The blow has been struck—the walls have been levelled with the earth—an open breach has been effected into the very heart of Russia—and the inroad of modern opinions and freedom of thought through the yawning gap is inevitable and irresistible. When the Allies landed at Old Fort, on the 14th of September, 1854, they took possession, not of Muscovite territory, but of the Muscovite mind.*

* The *Czas*, the Austrian journal of Cracow, says:—"In the night of the 20th ult., the recruitment of 30,000 men, from the age of nineteen to thirty-five, took place in Poland." This is the most terrible form of serfdom extant. But the Russian government are already alive to the pressure of the times. A university is to be founded at Nicolaieff. An observatory—arranged for meteorological as well as astronomical records—is also to be erected in the city. Proposals for railway undertakings are already in the market. These reports, if true, speak volumes. The following is from the *Times*' Special Correspondent, April 4th, 1856:—

"The demolition of trenches, works, and houses in the city continues daily and incessantly, so that the south side will soon be as desolate and ruinous as Thebes or Palmyra. Every hour long trains of men pass by with beams of timber and

France, as a great military power, is the first and foremost of the European family. She has never wanted a great statesman nor a great warrior, at her command, or on her throne, from Pepin the Little down to Napoleon the Great. At the same time, she is the most fickle and the most constant, the bravest and the lightest hearted, the most ingenious and the least persevering, the most enthusiastic and the most frivolous, the most erudite and the most superficial, of the chief western powers. Her history abounds with the saddest and the most joyous of anecdotes and annals. The Merovingian, Carlovingian, and Capetian dynasties are full of characters as remarkable for their piety as for their vices, for their debaucheries as for their saint-like virtues. It is a tale of romance from first to last, and never palls upon the taste with dullness and inaction. Her chivalry is proverbial. For the sake of liberty, real or imaginary, as it may be, she has changed her dynasty and its titles, her ensigns and her flag, more than once within the memory of some of the present generation. She has been infidel and Christian with the same breath; she has deposed and defended the head of her church in the course of the last half century. Her spirit has planks on their shoulders, which are taken out of the remains of the White Buildings. Had fire been rained down from heaven on the devoted city, its annihilation could not have been more complete. The stranger who halts to survey it from the neighbouring heights, deceived by the whitewashed and plastered walls of the houses, might think that Sebastopol was still a city; but when he walks through its grass-grown, deserted streets, formed by endless rows of walls alone, of roofless shells of houses, in which not one morsel of timber can be seen, from threshold to eaves; when he beholds great yawning craters, half filled with mounds of cut stone, heaped together in irregular masses; when he gazes on tumuli of disintegrated masonry,—once formidable forts, and now shaken, as it were, into dust and powder; when he stumbles over the fragments of imperial edifices, to peer down into the great gulfs, choked up with rubbish, which now mark the site of the grand docks of the Queen of the Euxine; and beholds the rotting masts and hulls of the sunken navy, which was nurtured there; when he observes that what the wrath of the enemy has spared is fast crumbling away beneath the fire of its friends, and that the churches where they worshipped, the theatres, the public monuments, are specially selected for the practice of the Russian gunners, as though they were emulous of running a race in destruction with the allied armies,—he will no doubt come to the conclusion that the history of the world affords no such authentic instance of the annihilation of a great city. It is hard to believe that the site can ever be made available for the erection of houses or the construction of docks; but I am by no means certain that the immense resources in the command of manual labour possessed by the Government of Russia, of which this very struggle has afforded us all such striking proofs, in the Quarantine Battery, the Bastion Centrale, the Bastion du Mât, the Redan, the Mamelon, and the Malakhoff, may not be available in time to clear away these modern ruins, and to rebuild houses, theatres, palaces, churches, forts, arsenals, and docks as before. To prevent any successful attempt to use the old materials in the docks, our engineers are now busy in destroying the coping-stones of granite and the larger masses of stones in the masonry; but in the Inkermann ravines there are inexhaustible supplies of building material, which can be floated by the Tchernaya into the waters of the harbour with very little trouble. The immense quantity of cut stone lying in piles at the upper end of the harbour shows that the allies interrupted the Russians in been subtle in the cause of Christianity ever since she was first called Frank. Warmly attached to her religion, which she never at heart renounced, she has endeavoured to propagate it all over the world. Her missionaries have always supported a high reputation in the most distant quarters of the globe. She alone owns the splendid victory over the Saracens, in the eighth century, which so effectually freed Europe from their grasp. A thousand years ago, the empire of the Franks was the most powerful state in Europe; and for a long period she was the centre of the civilized world. To her the student owes a willing debt of gratitude for her unrivalled works in science and literature, modes of feeling and sound logic; and the scholar and man of taste thanks her for so much that is beautiful, attractive, and instructive in the fine arts. Often buried beneath the agitated surface of external events, her intellectual progress has never ceased, and her history forms an essential and magnificent theme in the life of every civilized community. The dead monotony of the Byzantine court expired in a decreasing scale of moral, political, and intellectual degradation, and the Saracenic sway was but the hasty growth of circumstances unable to survive its the development of the splendid architectural plans which it was the ambition of emperors to accomplish, and which had engaged every thought and energy of the Muscovite governors of the Crimea. The shells of princely mansions which remained on the French side of the town have been battered to atoms by the Russian batteries on the north side; the theatre has been demolished, and the beautiful church of St. Peter and St. Paul laid in ruins by the same implacable foe, and they have directed particular volleys of round shot and shell on a monument to one of their naval heroes, which stands conspicuously placed in front of a beautiful little kiosk in the midst of a garden, to which there was a fine approach from the place behind Fort Nicholas by a handsome flight of steps, now knocked to pieces. On a quadrilateral pedestal of some pretensions, supporting entablatures with allegorical devices, and ornamented at the summit by a *puppis*, were inscribed when first I saw it the name of "Kazarski," and the dates 1829 and 1834, with an intimation that the monument was erected to posterity in his honour. Most of the letters have been stolen and knocked away now; and had not the fire from the north ceased, the pedestal itself would have disappeared likewise. The French garrison, somewhat harassed by the incessant fire on the town, which, however, did them or us but little mischief, have constructed out of the *débris* of the houses a very neat *quartier* inside the walls, which is altogether new, and presents a very strange appearance from its contrast to the ruins around it."

The subjoined is an admirable description of the Muscovite, Tartar, or Mongolian physiognomy. *Times*' Special Correspondent, April 4th, 1856:—

"There is a wonderful family likeness among the common soldiers. The small round bullet head, the straight light hair, high cheek bones, gray keen eyes rather deeply set beneath straight and slightly-defined eyebrows, undemonstrative noses with wide nostrils, large straight mouths, square jaws, and sharp chins are common to the great majority of them. Their frames are spare and strongly built; but neither in stature or breadth of shoulder do they equal the men of our old army of 1854. Many of the officers are scarcely to be distinguished from the men in air, bearing, or dress, except by the plain, ill-made, and slight swords which they carry from an unornamented shoulder-belt; but now and then one sees a young fellow with the appearance of a gentleman, in spite of his coarse long coat; occasionally a great tall lumbering fellow, who seems to be of a different race from the men around him, slouches along in his heavy boots."

own internal distractions. But the French have, in spite of some dark exceptions to the contrary, exhibited the gradual organization of a Christian state, and the slow development of Christian science, for upwards of ten centuries, and they are as young now in valour and spirit as they were when Clovis held the sceptre and bowed his haughty head before St. Remy at the font, upon his conversion to Christianity, supposed to have been granted to the prayers of his sainted wife, the fair Clotilda.*

Such are the opposite characters of the two nations that have lately confronted each other in the field, or during the weary siege. When gun was pointed at gun, and trench was dug, and rampart raised against counter rampart and counter trench, how little did the well-disciplined officers who headed the charge, defended the breach, or led the assault, fancy, as they dropped at the cannon's mouth or fell pierced with the sword or bullet, think that they were only fulfilling the destiny of nations, and exemplifying the distinction of races. Their fate will serve to illustrate some curious questions in ethnology, or settle a worn-out date in a doubtful point of history: Sebastopol fell on the 8th September, 1855, and a treaty of peace was concluded at Paris, March 30th, 1856.

In the journals of the day, relating to the peace, there is an air of languor that reminds us of a person that has been overfatigued. It seems to be a feeling of relief at having been allowed to lay aside a burden beyond his strength. Nor is this sentiment peculiar to this country, for, although expressed in a different manner, it is perceptible on the other side of the channel. The eagerness with which the first proposals of peace were met by the Continental powers is, if possible, more undisguised than

* *Times*, March 7, 1856:—

"But in France government is neither founded on prescription, as with us, nor on superstition, as in Russia. The qualities which secure obedience in France seem now to be purely personal, and little is gained by birth, unless it be united with those qualities which conciliate the respect and compel the obedience of mankind.

"The lessons of history on this subject are so exceedingly striking and appropriate that it is impossible for an impartial writer to consider such an event as the present without alluding to them. And yet, if we were permitted to dwell in the land of hope rather than in that of reality, how gladly would we believe that in the birth of this infant, at the very moment that gives renewed peace to Europe, we find a pledge for the termination of those incessant convulsions which, from the assembly of the States-General under Louis XVI., have, at longer or shorter intervals, never failed to agitate the Government and people of France! Happy indeed will be the destiny of Louis Napoleon if he succeed, not only in founding his own power on a secure basis, but in transmitting it unimpaired to a son who may inherit the talents of his father, while free from the difficulties and dangers which beset his early path, and raised him only after long suffering and severe discipline to a position in which he has upheld the material interests of France with one hand, and nobly asserted her dignity and pre-eminence among the nations of Europe with the other."

the want of enthusiasm on our part. The English were alive to the fact of their resources being equal to a second, or even a third, campaign, and of the strong probability of their coming out of the last battles far more victoriously than from the first. Nevertheless, they were willing to decline any further contest, and were content to retire in full force behind the bulwarks of their own defences. But it is, also, evident that Russia was exhausted, if not used up, and France, from whatever cause, only too eager for peace. But whether on their side or on ours, two short years of warfare have been enough to damp the warlike ardour of the combatants. It is useless to plead the milder temper of the present age: the truth is, the burden was too enormous to be borne any longer without danger to the whole of Europe.*

The next power is that of the Turkish Empire, whose interests we have espoused, and with whom we have enlisted ourselves. But it is not the first time that the Turks and Christians have fought together. They were united in the reign of Justinian, in the sixth century, and in that of Heraclius, in the eighth; and then there was the famous alliance of the Sultan Solyman with Francis the First of France, in the sixteenth. But none of these alliances lasted long. Even the cunning treaty of commerce entered into by Venice with Mohammed II., which brought down upon the Venetians the hatred of Christendom, was of a very brief duration. The disciples of Mahomet do not approxi-

* *Times*, April 2, 1856. Correspondent from Paris :—

"We have already learned by the telegraph how the news has been received in London. I believe I am not in the slightest degree mistaken when I state that the best feeling prevails here among all classes, and almost all parties, at the conduct of England throughout. No one knows better than this people that if there ever was a time when England was prepared to carry on war with vigour, and with all the elements of success, it is the present; that her army is in courage, discipline, experience, and resources such as it has seldom been, and that her maritime force is unexampled, even in her own history. They know, too, now that passion has calmed down, that England has not entered into the present, or rather late war, for selfish motives, and that she was prepared to continue it, not out of any inordinate love for war, any more than for any projects of ambition, but to obtain an honourable peace, which, as the Emperor very properly said, does not inflict humiliation on any one, while it secures for a long period the tranquillity of Europe and the independence of every European state. They know too that the feeling which influenced England was a far purer and a higher one than the vain longing for military glory; and that, while they are proud of having drawn the sword in a just and noble cause, they are wise enough to know when that cause is saved, and moderate enough to be content with having saved it. When, after all, one reflects for a moment on all that Russia once demanded, and all that she has now given up, the peace that has just been concluded can hardly be pronounced other than glorious in its results; and on a calm consideration of all that has occurred since the British and French flags first floated in the Euxine, the man must, in my opinion at least, be unreasonable indeed if he be dissatisfied. Here it is not anticipated that such will be the case, but that in England, as in France, the peace that has been concluded will be found honourable for all concerned, and, because honourable, satisfactory."

mate to the followers of Christ in any one of their relations. They never have agreed, they never can, and they never will. They are inherently inimical to each other. Our institutions, laws, marriage, mode of government, course of civilization, style of thought, modes of intercourse, habits, dress, and behaviour, are diametrically opposite. As a people, they are immiscible, unapproachable, and antagonistic with ourselves. *We* cannot change, neither can *they*. *They* are Asiatics, *we* are Europeans. We are all energy and adventure—they are all apathy and fatalism. They are to-day precisely what they were in 1454—that is to say, a Tartar camp pitched on the borders of Europe. Hence it has happened that war between us and them is but a matter of course, while peace is a diplomatic fiction, which can continue only so long as it serves the nonce.*

Had the policy which dictated the Crusades been persisted in for one century longer, not a turban nor a scimitar would have been left on this side the Bosphorus and Dardanelles. They who suppose that the Crusades were nothing more than a Quixotic exploit for the purpose of gratifying an unmeaning spirit of devotion and chivalry, know but little of history. The Crusades, as far as they went, were the salvation of the West; their only fault is that they did not go far enough. The cause that produced them was a stern necessity of the last importance to mankind; and as the late expedition to the Crimea has checked the inroads of Russia upon Europe, so the Crusaders effectually repulsed the invasions of the Saracens and Turks from the East. As far back as the ninth century the Saracens nearly made themselves the masters of Rome and the whole of Italy. Had they succeeded in their attempt, resistance would have been in vain, and the ascendency of Islamism in the Western hemisphere would have been complete. The Crusades were the only means left for turning the enemy's flank, by descending upon Asia

* *Times*, April 5, 1856 :—

"By the war of 1853 all former treaties with Russia were abrogated. More than one of those treaties had defined the position of the Principalities. The suzerainty of the Sultan—the administration by Hospodars, in the last instance chosen for a term of seven years—the protectorate of Russia—the restriction on the entry of Turkish troops, were all laid down in treaties commencing in the last century, and coming down to the modern days of 1812, 1829, and 1849. All former customs were abolished by these documents, so that it seems probable that, according to Grotius and his brother writers, the Sultan must, now that the treaties themselves are abrogated, resume his rights, 'pure and simple,' with absolute authority. It is this important matter which still remains to be decided by the wisdom of Europe. The Principalities, as the debateable land of the East, with a rich soil, the finest water-carriage in Europe, and a population unwarlike, and capable of being made industrious, is just the prize for which military monarchies are likely to contend. Moldo-Wallachia is now free from the Russian protectorate; it must shortly be withdrawn from Austrian occupation. What is then to follow is the problem for statesmen to resolve."

itself, and carrying the war into the heart of *their* land, instead of suffering them to invade *ours*. The Crusades, therefore, were the result of a policy the most enlightened and far-sighted of its kind, and it was well nigh brought to a triumphant close on the 7th of October, 1574, when Don John destroyed the Turkish fleet in the Gulf of Lepanto. That immortal day broke the Ottoman pride, and undeceived Europe, which fancied that until then the Turkish fleets were invincible.*

And now as to ourselves. The proverb says it is easy enough to praise the Athenians at Athens; and if we extol our native land, where is the patriot who shall blame us? But let us be candid. Let us look down upon our country from the highest point of sight, and scan its merits, if not its demerits, with the eye of an impartial philosopher.

In the present state of public opinion, with a Reform Parliament, and the great principle of religious toleration no longer a question in abeyance, but a positive agent alive and alert in the bosom of the Cabinet itself, it is impossible that any ministry, formed upon whatever conditions it may be, can hold together for any length of time, unless it act in accordance with these popular and acknowledged data in politics. Trade and intercourse with foreign nations is no longer on the same footing that it was only twenty years ago. Monopoly is at an end;—the free trade with China shows this. Commerce cannot be any longer shackled and restricted by fetters, which, while they gall the many, aggrandize the few. Public opinion is not to be passed over as a mere sentiment of no force, except when it coincides with the policy of cabinets, the prerogatives of princes, or the maintenance of national egotism. The opinions of many are the voice of one—the mind of the ignoble and the pauper is as energetic as that of the wealthy and the noble. The handicraftsman owns a private judgment and a free will as clear and discerning as that of the statesman. The private interests of the world are common property, which can no longer be molested with impunity, nor excluded without opposition from a fair participation in their proper share of the public welfare. The prime minister of the present day must have the courage to face the whole world, and the wisdom to discern that, while it is his first duty to serve his sovereign, it is, at the same time, his most obvious policy to answer the requests, to meet the wishes, to supply the wants, and to ameliorate the sufferings of the totality of mankind. Party is

* Cervantes was wounded in the battle of Lepanto. (*Don Quixote*, part 1, ch. xxxix. Madrid, 1799, 16mo, tome iv. p. 40.) In the opening of the 2nd part, Cervantes recurs to this famous battle (*ibid.*) with expressions of the greatest warmth. Lord Bacon, in the dialogue *De Bello Sacro*, wonders that Don John was never canonized.

at an end. The watchwords of Whig and Tory have lost their meaning. A new designation is wanting to signify the precise character of Great Britain's line of conduct at the present epoch. During the last quarter of a century the British constitution has undergone a revolution, bloodless indeed, but not less portentous to her future destinies, than was the Reformation by Henry VIII., or the invasion of these shores by William the Conqueror.

The entire repeal of the Corn Laws was but the touchstone to a set of ideas that must eventuate in free trade altogether, just as the Reform Bill was but the overt act of another train of ideas respecting popular liberty, which must eventually end in a modified republic. And so, likewise, the removal of religious disabilities was the act of a great-minded people, proclaiming that a change had passed across the spirit of the age, and put itself at the head of civilization; it dispersed the darkness of the middle ages by acclamation, and showed that it could be religious without bigotry, and right-minded without superstition. It was a noble deed that penetrates to the inmost recesses of the heart. All these questions have come upon us with giant strides, and it is already manifest that their issue is, as far as it has gone, entirely beneficial to the well-being and advantages of the people by whom they have been brought about. Their intelligence and good sense is known to all the world; and our stability in the midst of the revolutions of 1848 is a solid proof of this. England must go forward, for she cannot go back; nay, more, she *is* going forward, and *will not* go back.

Her position as a maritime power, both naval and commercial, is the mightiest the world has ever yet seen. Her colonies are distributed all over the globe; her trade is settled in every port; her flag flies on every sea; her personal bravery is undisputed; her navigation unrivalled; her liberty, both national and individual, large and secure; and the freedom of her press uncompromised, *uncensored*, and unabused. There is no doubt that a population of this description must be powerful, because of its intelligence, and cannot be conquered, because it is not only free itself, but also seeks the freedom of all others. Such is the fourth power recently engaged in the war.*

* *Times*, April 2, 1856. Correspondent from Paris:—

"In my letter of yesterday I mentioned that immediately after the signing of the treaty of peace at the Ministry of Foreign Affairs, the plenipotentiaries proceeded to the Palace of the Tuileries, to communicate the important fact to the Emperor in person. His Majesty received them in the Salon des Ambassadeurs, attended by the officers of his household. When the news was announced, the Emperor is said to have expressed his thanks to the plenipotentiaries for having come in person to him with such agreeable tidings. He observed that the result of their labours during the conferences was the complete realization of the speech delivered by Lord Clarendon in the House of Lords, and that the peace which the allies were determined on concluding was one which carried with it no humiliation to Russia,

The little kingdom of Sardinia, which has played so heroic a part in the contest, ought not to be passed over in silence, without receiving its due meed of praise; and the mention of Sardinia leads us to think of mismanaged Italy, whose fortunes are now trembling in the balance.* Another congress may have to determine questions whose explosive elements may ignite at a touch, and set the whole of Europe once more in a blaze. It is an instinctive feeling of uncertainty concerning points of this import which cannot be included within the proposed peace, that hangs heavily on thoughtful and foreseeing men, besides the deep and universal consciousness that peace is not the ordinary state of affairs, any more than health is the usual condition of life, or prosperity the rule of fortune, but that, on the contrary, prosperity is liable to be interrupted by reverses, health by disease, and peace by war.

And, after all that has been done, what have we gained by our huge exertions? After the waste of half a million of lives, a hundred millions of money, the destruction of our foe's choicest strongholds,—Sweaborg and Sebastopol,—the sinking of his ships, the capture of his small craft, the petty marauding of his coasts, and the display of our own prowess, what good have we accomplished for ourselves or for the world? Is the world advanced one inch nearer to happiness, freedom, and stability? Is Europe wiser and better than she was two years ago? Is France a greater country, England a more flourishing community, or Russia a less formidable adversary? We pause for a reply; but time only can answer these questions. A story is told of Talleyrand, when he was old and confined to his easy chair, listening to a popular tumult in the streets of Paris. To the sound of the tocsin and the discharge of the musketry he beat the tattoo on the table before him, murmuring to himself the words "Nous triomphons!" "Who is victorious?" asked those and which did not compromise the dignity or independence of any one; it was, in fact, such as a great nation might propose or accept without degradation, and it therefore had all the elements of solidity and durability; and he added, that so favourable a result was, in a great measure, owing to the conciliatory spirit and moderation which marked the policy of England, and which was particularly felt in the course of the present conferences."

* *Times*, April 8, 1856:—

"To a reported exclamation of the French Emperor, of 'What can one do for Italy?' Count Cavour has answered by a memorial which states the principle grievances of Italy in general, as well as of the individual States. The Milanese and Venetian territories, the Papal States, the kingdom of Naples, all suffer from different forms of the same malady.

"Taking it for granted that there must be for a long time to come a struggle between the liberal and absolutist principles in Europe, Sardinia is anxious to range herself and her sister States on the side of freedom, as represented by France and England. Austria she considers as only temporarily and by chance the opponent of the Czar."

about him. "Never mind who," replied the wary diplomatist; "never mind who wins or loses—we shall learn that to-morrow!" And this is the gist of the whole matter; for, in the course of ages, it is of little consequence whether this emperor is defeated or that emperor conquers, or this people is supreme, for the space of a year, a lustrum of years, or a brief century. The end is the same:

> "They strut and fret their hour upon the stage,
> And then are heard no more."

But no great event ever happens without a cause proportioned to its greatness. What was the cause, then, for which we so liberally opened our purses, and so resolutely ventured our lives? It was simply to hinder one neighbour from breaking into the house of another neighbour, and robbing him of his goods. So far as this was the case we have succeeded in our plans, and, considering what the chances of success are, this is affirming a great deal. But something more was aimed at beyond the object we started with the intention of reaching; and should this ulterior point of sight have been, in fact, reached, and should its attainment prove to be a permanent one, it was certainly worth the blood and treasure expended upon it.

If there be anything real in this world it is the spirit of Christianity. The mutual fellowship of mankind upon an equal footing, and subject to equal laws, is the rule of government, the end of civilization, and the climax of humanity. There is no doubt that the late conflict has achieved a grand desideratum in this respect. It has already led to kindlier feelings between the eastern and western nations, a reciprocity of rights, a concession of prejudices, and a toleration of creeds. This sentiment is also expressed by Russia herself, and evinced in some of her late acts; and it cannot fail to be pressed still more closely upon her attention by the force of circumstances, that speak with too loud a tongue not to be listened to. An easier intercourse with countries, hitherto all but hermetically sealed against our entrance, is another necessary result of the past contest, together with a greater freedom of thought, a quicker circulation of ideas, an exchange of literature as well as of more substantial commodities, and last, though not least in the order of society, intermarriages, fresh blood, new connexions, new manners, new customs, and new things. These benefits will be felt by all for a season at least, if not for a continuance; and Russia, in particular, will be more known to us than she has been before, and we to her in return. A better understanding among all parties must ensue, and a new order of affairs arise out of the old. It is but a repetition of the effects of more extensive emigrations we read of in former times, such as those of Sesostris or Cyrus, Cæsar or Pizarro. It is a passing panorama replete with imagery, and we ourselves are taking part in one of those striking scenes in the drama of nations, which will remain emblazoned on the page of history to all generations.

And here we must rest. The sun is declining calmly over the waters of the Black Sea, and shedding its oblique rays on the remains of what was once Sebastopol. The waves that heave upon its coast, or ripple upon the surface of its placid harbour, are reflecting the golden hues of evening. Everything is still, and the more solemn from the quietude that covers the dead, the ruins of the fortress, the long-cherished hopes of the czars, the camps of the allies, and the outposts of the Russians along the opposite heights. All is still: not a gun nor a rifle disturbs the deep repose; only a bittern booms from the sedgy ground* of the Tchernaya, or a vulture hovers over an unburied skeleton in the valley of Inkermann, or a stray dog bays at its own shadow against yon broken wall. It is the stillness of death. They who fell in action have long since passed to their last account; and the

* *Times*' Special Correspondent, April 4, 1856 :—

"Further back in the sedgy ground are lynx-eyed duck-hunters, plunging through the wavering bulrushes, and knocking over the flat billed swimmers with an incessant pop which puts one in mind of the old sounds from the rifle pits on a quiet day in the trenches. Some of these grey-coated gentry—the Ruskies, not the ducks—are wonderful shots, and may be seen carrying away strings of wild fowl over their shoulders towards Mackenzie's Farm. The French, on our side, are as assiduous, but by no means so successful in pursuit of game, which, indeed, is scarce towards the western bank, and now and then, to the indignation of our more scrupulous and better disciplined sportsmen, they cross over the river and wage with the Russians a common war against *anatidæ, scolopidæ,* and *gallatores.* When I say 'better disciplined,' I mean only to imply that the severities of our chiefs, who threaten any officer who may be found out of bounds with the penalties of a court-martial, deter them most effectually from taking the liberties in which our allies so gracefully indulge. 'Have you killed anything?' said I to a gallant young Guardsman, knee-deep in slush. 'No ; these confounded —— frighten them all to the other side, where they are so thick they can't be missed, and then they go over and shoot them like sparrows, while we poor devils are kept here and will be broke by old C———y if we follow them.' However, wild ducks have been killed and eaten by us, and the pintail and the teal, the golden-eyed pochard, the widgeon—his tufted brother, the little grebe, and some other varieties, have undergone the trying operations of the British *cuisinier.* As we ride along, lo ! a fusillade springs up in the marsh, and grandly through the sky, in dazzling relief against its azure, sail two milk white swans, with outstretched necks and black bills, cleaving their way against a strong east wind, and jerking a wing now and then in acknowledgment of some high-flying bullet that has just gently tickled the feathers of their snowy mail. Then up rises a train of herons, or a noisy comitatus of brent geese, or a flight of mallard and duck, with whistling wings, or heavy bitterns, or agile snipe and cloudy streaks of plover, and distract the attention and the aim of the excited pot-hunters. For several long miles this active chase goes on under the solemn brow of Inkermann, past the deep gorges of these blood-stained ravines, by the deserted City of Caves, the dwelling-places of mystic and forgotten races, till the Tchernaya, expanding as it flows, gains on the yielding earth, and eats its way with many mouths through the fat *marais* into the blue waters of the roadstead of Sebastopol."

largest army* that England ever counted in the field is preparing to return, crowned with laurels, to its native land. May the peace that they, together with our allies, the gallant French and the brave Sardinians, have achieved for us, be as lasting as their valour has been unfailing and their arms triumphant. We linger in fancy over this remote corner of the earth, where lie buried so many whom we loved and honoured, and from whence has sprung that glorious end for which alone they fought, bled, and conquered!

* *Times*, April 8, 1856:—

"We have to keep that army in efficiency, if not in full numbers, and, should it suffer any decay, should it crumble into regiments, should it disappear in country quarters and colonial stations, and have no aggregate existence except in the monthly list, showing whereabouts in the world each hundredth part of the army happens to be buried, we are sure that the Government of this country will be held responsible for the calamities certain to fall on us in the next great war."

